# The Learning Curve of Endoscopic Lumbar Interbody Fusion: A Systematic Review

**DOI:** 10.3390/jcm14248926

**Published:** 2025-12-17

**Authors:** Yong Ahn, Hajin An, Sol Lee, Hee Seon Choi, Hye Soo Rho

**Affiliations:** 1 Department of Neurosurgery, Kyung Hee University Hospital at Gangdong, Kyung Hee University College of Medicine, Seoul 05278, Republic of Korea; cescjin@gmail.com (H.A.); hwengel@naver.com (H.S.C.); rorocap@hanmail.net (H.S.R.); 2 Biobytes Inc., Seoul 26460, Republic of Korea; iamleesol@gmail.com

**Keywords:** endoscopy, learning curve, lumbar vertebrae, spinal fusion, spinal stenosis

## Abstract

**Background/Objectives**: Endoscopic lumbar interbody fusion (ELIF) represents a key milestone in minimally invasive spinal surgery, offering reduced tissue trauma, lower complication rates, and faster recovery compared with open fusion. However, its steep learning curve remains a major barrier to widespread adoption. This systematic review aimed to synthesize current evidence on the ELIF learning curve and identify factors that influence the acquisition of surgical proficiency. **Methods**: A comprehensive literature search of PubMed, Embase, and the Cochrane Library was conducted for studies reporting quantitative analyses of the ELIF learning curve. Eligible articles included clinical data describing operative performance, complication rates, and learning curve cutoff points. Study quality was evaluated using the Newcastle–Ottawa Scale. Pooled data were analyzed to determine the mean cutoff point between the early and proficient phases and to compare outcomes across surgical approaches. **Results:** Five eligible studies encompassing 425 patients were included. Operative time was the most frequently assessed outcome, followed by hospital stay and complication rates. The pooled cutoff point for operative time was 23.4 ± 8.9 (range, 12–29) cases. Full-endoscopic ELIF tended to require longer operative times but resulted in shorter hospital stays than biportal techniques. **Conclusions**: ELIF reflects the evolution of endoscopic fusion techniques. The proficiency threshold varies according to the outcome parameters and the type of endoscopic system. Structured training programs and standardized educational pathways are essential for optimizing the learning process and ensuring safe and efficient implementation.

## 1. Introduction

Minimally invasive spinal surgery has gained attention as a key advancement in the treatment of spinal disorders. For example, endoscopic spine surgery has emerged as one of the most representative and transformative approaches of minimally invasive surgery, offering minimal tissue injury and serving as an effective alternative to traditional open procedures [1,2,3,4,5,6,7].

The indications for endoscopic spine surgery in the management of degenerative spinal disorders have broadened considerably over time [8,9]. While endoscopic decompression has long been the principal focus, recent advances have extended the use of endoscopic techniques to fusion procedures. To overcome the limitations associated with conventional spinal fusion, various minimally invasive lumbar fusion techniques have been introduced [10,11,12,13]. Among these, endoscopic lumbar interbody fusion (ELIF) has rapidly emerged as a state-of-the-art technique, offering substantial clinical advantages and ranking among the most promising contemporary spinal surgery techniques [14,15,16,17,18,19,20,21,22]. Learning curve analyses have been widely applied across other surgical disciplines, including laparoscopic abdominal surgery and arthroscopic procedures [23,24,25]. Systematic reviews in these fields consistently show that increasing case volume leads to improved operative efficiency, reduced complication rates, and shorter hospital stays. These findings highlight the importance of understanding the learning process in minimally invasive spinal surgery and provide a strong rationale for evaluating the learning curve associated with endoscopic lumbar fusion. ELIF combines the advantages of traditional endoscopic techniques such as preserving normal tissues, reducing surgical morbidity, and achieving faster recovery, with additional benefits specific to fusion procedures [26,27,28,29,30,31,32,33]. ELIF offers precise visualization of the endplate during preparation, enabling selective removal of the cartilaginous layer while preserving the intact bony endplate, which may reduce postoperative subsidence and improve fusion rates. These features make ELIF a highly advantageous approach for treating degenerative spinal conditions compared with conventional open lumbar interbody fusion. Despite these significant advantages, the steep learning curve of endoscopic spine surgery remains a critical barrier to its widespread adoption by standard spine surgery societies [34,35]. The complexity of learning this endoscopic technique poses challenges for spinal surgeons, particularly during the early stages of the learning process during their residency or fellowship.

We systematically reviewed published studies on the learning curve of ELIF to analyze the characteristics that determine the cutoff point for proficiency level and to discuss strategies for improving the learning process and facilitating broader implementation of this advanced surgical technique. We further compared the learning processes for full-endoscopic and biportal ELIF. To the best of our knowledge, this is the first comprehensive review and analysis of the learning curve for endoscopic lumbar fusion techniques.

## 2. Materials and Methods

### 2.1. Search Strategy

This study, which was exempt from review by our hospital’s institutional board, was conducted with the utmost integrity, following the principles of the Declaration of Helsinki. Two reviewers searched the PubMed, Embase, and Cochrane Library databases to identify clinical articles and studies involving learning curves in ELIF surgery. A literature search was conducted from inception to 27 January 2025, using the following terms as text words or Medical Subject Headings (MeSH) terms. Detailed search strategies for each database are provided in Supplementary Materials Text S1. This systematic review was conducted in accordance with the Preferred Reporting Items for Systematic Reviews and Meta-Analyses (PRISMA) 2020 guidelines. The completed PRISMA checklist is provided in Supplementary Materials Table S1. The review protocol was not registered in PROSPERO or any other registry because it was based on previously published data and did not involve direct human participation.

### 2.2. Search Strings

For PubMed:

((“biportal endoscopic”[TIAB] OR “uniportal endoscopic”[TIAB] OR “full-endoscopic”[TIAB] OR “unilateral biportal endoscopic”[TIAB]) AND (“endoscopic”[TIAB]) AND (“lumbar”[TIAB] OR “lumbosacral”[TIAB] OR “thoracolumbar”[TIAB]) AND (“interbody fusion”[TIAB] OR “Spinal Fusion”[Mesh])) AND (“learning curve”[TIAB] OR “learning curves”[TIAB] OR “training curve”[TIAB] OR “training curves”[TIAB] OR “Learning Curve”[Mesh]).

For Embase:

(“biportal endoscopic” OR “uniportal endoscopic” OR “full-endoscopic” OR “unilateral biportal endoscopic” OR “endoscopic”) AND (“lumbar” OR “lumbosacral” OR “thoracolumbar”) AND (“interbody fusion” OR “spine fusion”/exp) AND (“learning curve” OR “learning curves” OR “training curve” OR “training curves” OR “learning curve”/exp).

For Cochrane Library:

((“biportal endoscopic”[TIAB] OR “uniportal endoscopic”[TIAB] OR “full-endoscopic”[TIAB] OR “unilateral biportal endoscopic”[TIAB]) AND (“endoscopic”[TIAB]) AND (“lumbar”[TIAB] OR “lumbosacral”[TIAB] OR “thoracolumbar”[TIAB]) AND (“interbody fusion”[TIAB] OR “Spinal Fusion”[Mesh])) AND (“learning curve”[TIAB] OR “learning curves”[TIAB] OR “training curve”[TIAB] OR “training curves”[TIAB] OR “Learning Curve”[Mesh]).

### 2.3. Literature Selection Criteria

This study included: (1) articles describing the ELIF procedure with quantitative learning curve data, including clinical outcomes and cutoff points or asymptotes of the learning process; (2) level of evidence of I, II, III, or IV; (3) studies involving human patients; and (4) articles written in any language. Studies describing the retroperitoneal or lateral approach, simple case reports, technical notes, editorials, letters to the editor, comments, narrative reviews, or animal studies were excluded.

### 2.4. Data Collection

The contents of the studies, including the title, abstract, and full text, were screened stepwise. Discordant opinions at any stage of the screening process were resolved through discussions between the reviewers. Studies for which all reviewers agreed to their eligibility were included. Data were collected independently from the selected studies and organized through a reviewer discussion. Various parameters were recorded: Publication date; study type; surgical style; number of patients; demographic data; preoperative conditions; operative data, including operative time, blood loss, and intraoperative events; and postoperative outcomes including pain scores, functional status, complications, and revision surgeries. Patients were divided into early and late groups based on the cutoff point or asymptote of the learning curve. The cutoff point for technical proficiency was defined as the number of surgeries in which the learning curve plateaued.

### 2.5. Quality Assessment

Quality assessment was performed using the Newcastle–Ottawa Scale (NOS), which was developed to evaluate the quality of non-randomized clinical trials, including cohort and case–control studies [36,37]. It consists of eight items divided into three domains: (1) selection of study groups, (2) comparability of study groups, and (3) outcomes in cohort studies or exposure in case–control studies. The overall quality of the studies ranged from 0 to 9. Stars were provided for each quality assessment item to provide quick visual assessment. Two reviewers independently performed the NOS quality assessment. Any disagreements were resolved through discussion, and when consensus could not be reached, a senior reviewer arbitrated. Inter-rater reliability for star allocation across the eight NOS domains was substantial (κ = 0.81).

### 2.6. Statistical Analysis

Statistical analyses, including meta-analyses, were performed using Review Manager version 5.3.3 (Cochrane Collaboration, Software Update, Oxford, UK). Outcome parameters were compared between early (learning) and late (learned) groups. The outcomes are depicted in forest plots with statistical estimates, 95% confidence intervals (CI), and relative weights represented by the middle of the square, horizontal lines, and relative size of the square, respectively. Continuous variables, such as operative time, pain scores, and functional outcomes, were weighted based on the number of patients, and the average weight was calculated. The heterogeneity of the study was determined using I^2^ statistics, calculated as I^2^ = 100% × (Q − df)/Q, where Q is Cochran’s heterogeneity statistic and df is the degrees of freedom [38]. I^2^ ranged from 0% (no heterogeneity) to 100%, with I^2^ >50% indicating heterogeneity. Based on the general criteria proposed by Higgins et al., I^2^ < 25% indicates low heterogeneity, I^2^ = 25–74% indicates moderate heterogeneity, and I^2^ > 75% indicates high heterogeneity [38]. Standardized mean differences (SMDs) were used because operative time, hospital stay, and other continuous variables varied in scale and variable measurement distribution across studies. The use of SMDs allowed for normalization of effect sizes and enabled pooled comparisons. When units were uniform and directly comparable, raw mean differences were also calculated as supplementary data. A random-effects model was employed due to methodological variations across studies (endoscopic systems, surgical techniques, and anesthesia types), as recommended by PRISMA guidelines for heterogeneous clinical data.

## 3. Results

### 3.1. Study Identification and Quality Assessment

**Figure 1:**
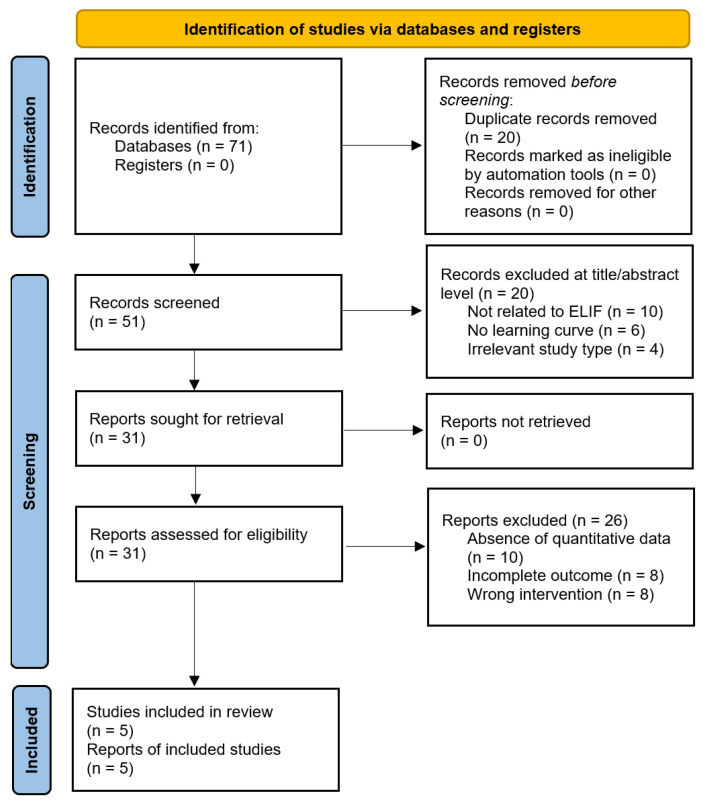
Flowchart of literature search and study selection.

Five studies that met the eligibility criteria were identified, providing a comprehensive overview of the research landscape [39,40,41,42,43]. A total of 71 records were identified through database searching. After removing 20 duplicates, 51 records were screened at the title and abstract levels, of which 20 were excluded for the following reasons: not related to ELIF or non-fusion endoscopic procedures (*n* = 10), absence of learning-curve or proficiency-related analyses (*n* = 6), and irrelevant study type (*n* = 4). The remaining 31 articles were subjected to full-text assessment. Twenty-six studies were excluded because of the absence of quantitative learning-curve metrics (*n* = 10), incomplete outcome reporting (*n* = 8), or the use of an incorrect intervention (*n* = 8). Ultimately, five studies met the eligibility criteria and were included in the final analysis. The complete screening process is illustrated in the updated PRISMA 2020 flow diagram (Figure 1).

The quality of each study was objectively assessed using the standardized Newcastle–Ottawa Scale. The final quality scores ranged from seven to eight stars, indicating good study quality (Table 1).

### 3.2. Characteristics of the Selected Studies

All selected studies were retrospective cohort studies that compared the early and late stages of the ELIF learning process with the posterior or posterolateral approach. The learning curves for both the cumulative sum test and the dichotomous comparison are depicted in all studies. The total number of patients was 425 (218 men and 207 women), with a mean age of 60.90 ± 10.82 years. Full endoscopic surgery was performed in two studies [40,42], and biportal endoscopic surgery was performed in three [39,41,43]. The approach method was posterolateral (transforaminal) in five studies [31,32,33,34,35,36,37,38,39,40,41,42,43] and posterior in one [40]. General anesthesia was used in five studies [39,40,41,43], and local anesthesia in one [42]. Most studies included single-level surgeries, except for one [39] that involved eight two-level surgeries. The mean operative time were 201.52 ± 97.22 min. The mean hospital stay was 7.66 ± 2.69 days. The overall fusion rate was 85.95% (318 of 370 cases). The overall complication rate was 5.65% (24 of 425 cases) and included dural tears, epidural hematomas, neurological symptoms, cage subsidence, and screw malposition. Substantial heterogeneity was noted across the included studies, including endoscope type (full-endoscopic vs. biportal), anesthesia, surgeon number, outcome definitions, grouping methods, and cutoff determination. I^2^-based heterogeneity assessments were added for all pooled parameters and the findings were interpreted accordingly. Table 2 summarizes the characteristics of individual studies.

### 3.3. Outcome Measures and Cutoff Points

**Figure 2:**
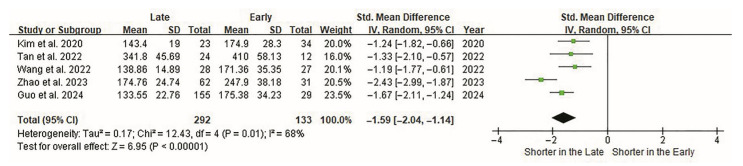
Forest plot of the operative time of ELIF. Operative times are evaluated in all five studies. Overall, 133 and 292 patients are included in the early and late groups, respectively. Forest plots present standardized effect sizes rather than raw units; therefore, operative time (minutes) is displayed as SMDs on the horizontal axis. The SMD between groups is −1.59 (95% CI −2.04 to −1.14). Operative time is significantly shorter in the late group (*p* < 0.00001). CI, confidence interval; ELIF, endoscopic lumbar interbody fusion; SMD, standardized mean difference.

The outcome measures included operative time, radiation time, estimated blood loss, hospital stay, pain scores, functional scores, complications, and fusion rate. The primary and most significant variable was operative time. The cutoff points determining the proficiency (learned) phase varied among studies with a mean of 23.40 ± 8.91 (range, 12–29) cases. The operative time in all studies was significantly short in the late group (*p* < 0.0001). Heterogeneity among the studies was moderate (I^2^ = 68%). Overall, there was a significant difference in operative time based on the cutoff point (*p* < 0.0001; Figure 2).

**Figure 3:**
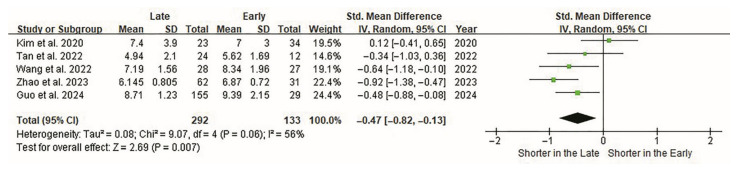
Forest plot of the hospital stay of ELIF. Hospital stays are evaluated in all five studies. Overall, 133 and 292 patients are included in the early and late groups, respectively. Forest plots display standardized effect sizes; therefore, hospital stay (days) is represented as an SMD rather than raw values on the effect-size axis. The SMD between groups is −0.47 (95% CI −0.82 to −0.13). The length of the hospital stay is significantly shorter in the late group (*p* < 0.007). CI, confidence interval; ELIF, endoscopic lumbar interbody fusion; SMD, standardized mean difference.

The length of hospital stay was the second most significant variable. In three studies, hospital stay was shorter in the late group than in the early group (*p* < 0.05) [41,42,43]. Heterogeneity among the studies was moderate (I^2^ = 56%). Overall, the cutoff points were significantly related to the length of hospital stay (*p* < 0.01, Figure 3).

**Figure 4:**
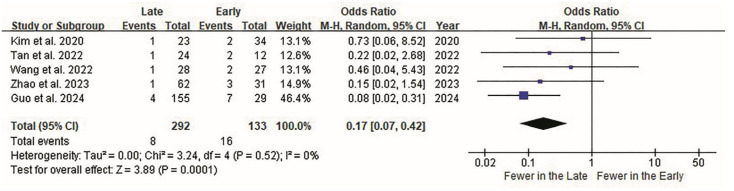
Forest plot of ELIF complications. Surgical complications are evaluated in all five studies. Overall, 16 of 133 patients (12.03%) in the early group and 8 of 292 (2.74%) in the late group report surgical complications. Complication rates are displayed as odds ratios (OR) on the forest plot effect-size scale, rather than raw event counts. The OR between the groups is 0.17 (95% CI 0.07 to 0.42). Overall, the complication rate is significantly lower in the late surgery group (*p* < 0.0001). CI, confidence interval; ELIF, endoscopic lumbar interbody fusion; OR, odds ratio.

Postoperative complications were the third most significant variables. Although most individual studies [39,40,41,42] showed no significant differences in the complication rates between the early and late groups, there was an overall difference in the incidence of complications. Overall, the complication rate was significantly lower in the late surgery group (*p* < 0.001; Figure 4). Although the study by Guo et al. [43] contributed the highest weight (46%) to the pooled analysis owing to its larger sample size and lower variance, the sensitivity analysis excluding this study confirmed that our findings remained robust. The re-analysis yielded a pooled odds ratio of 0.21 (95% CI, 0.08–0.55; *p* = 0.001), consistent in direction and statistical significance with the primary analysis, indicating that the overall conclusion is not unduly influenced by a single study.

Other variables, including radiation duration, estimated blood loss, pain scores, functional status, and fusion rate, showed no significant intergroup differences (Table 3).

### 3.4. Full-Endoscopic vs. Biportal Endoscopic

In ELIF, two endoscopic approaches are used depending on the type of endoscope used: the full-endoscopic approach [40,42], and the biportal endoscopic approach [39,41,43]. Operative time was shorter in the biportal endoscopic group, whereas the length of hospital stay was shorter in the full-endoscopic group. The mean operative time was 245.23 ± 89.42 min in the full-endoscopic group (*n* = 129) and 148.95 ± 34.46 min in the biportal endoscopic group (*n* = 296) (*p* < 0.0001). In the final stage (learned group), a significant difference was observed between the full-endoscopic and biportal groups. However, the magnitude of the difference in mean values was reduced compared to that in the early group (from 112.16 to 70.16 min), indicating that the gap in outcomes between the two approaches had lessened (Table 4). The mean hospital stay was 6.05 ± 1.38 days in the full-endoscopic group and 8.65 ± 2.55 days in the biportal endoscopic group (*p* < 0.0001). In the final stage (learned group), a significant difference remained, without a reduction in the gap between the groups (2.84 to 3.04 days; Table 4). The comparison between full-endoscopic and biportal ELIF was explicitly designated as a subgroup analysis, highlighting the technique-related differences contributing to heterogeneity.

However, both groups showed no statistically significant differences in other variables including pain scores, functional status, complications, or fusion rates. These findings were also observed even after mastering the surgical techniques.

## 4. Discussion

### 4.1. Cutoff Point and Outcome Measures of the Learning Curve

Typically, the cutoff point for distinguishing the early and late groups in learning curve studies can be determined using one of the two methods. The first method involves identifying the asymptote on the curve generated through cumulative sum analysis, providing a clear and objective measure. The second method allows for more flexibility as it is determined based on the researcher’s judgment or opinion. In this systematic review, the cutoff point was determined from the curve asymptote in three studies [39,40,41,42,43], whereas in two studies, it was defined using more flexible and arbitrary methods based on data analysis.

Three studies presented a comparative analysis of two groups [39,41,43], whereas two studies compared three groups in equal numbers [40,42]. However, a dichotomous comparison was fundamentally possible in all studies, with the first cutoff point playing a crucial role in guiding the focus and attention of the research.

Most studies presented a single cutoff point, averaging 23.40 cases, while one study reported different cutoff points depending on the outcome variables. Guo et al. [43] presented two cutoff points in the learning curve: 29 cases for mastering surgical skills and 41 instances for stable outcomes. This suggests that achieving a “learned” level may vary depending on the specific performance measure, sparking further interest and engagement in the research.

Although the outcome measures vary according to each learning curve study, the variables can be classified into two categories: (1) task-efficiency variables, including operative time, fluoroscopy time, estimated blood loss, amount of removed tissue, and number of hand movements, and (2) patient outcome measures, including surgical complications, pain score, functional status, revision surgery, patient satisfaction, fusion rate, and survival rate. Among the variables used, the most frequently used was the operative time, followed by the length of hospital stay. Operative time is a valuable metric for objectively and quantitatively monitoring the learning progress. However, measures of task efficiency alone, such as operative time or hospital stay, are insufficient to comprehensively evaluate surgical techniques. The true mastery of a surgical technique is most appropriately assessed through patient outcome measures, such as clinical success, complications, and surgical failure [44,45,46,47,48].

As the included studies were methodologically heterogeneous, the pooled cutoff points and complication rates should be interpreted as preliminary rather than generalizable findings. Given the small sample sizes and the heterogeneous and retrospective nature of the included studies, all interpretations of learning curve patterns in this review should be considered preliminary rather than definitive.

### 4.2. Full-Endoscopic Versus Biportal Endoscopic Approach

There were differences in surgical data depending on the endoscope used. The cutoff points indicating proficiency were 18.50 ± 9.19 cases for the full-endoscopic approach and 26.67 ± 8.74 cases for the biportal endoscopic approach. However, it is difficult to distinguish ease of learning based solely on this numerical difference.

The most important finding was that the full endoscopic approach tended to have a longer operative time but a shorter hospital stay than the biportal endoscopic approach. The differences in operative time and hospital stay between the full and biportal endoscopic approaches highlight the distinct advantages and tradeoffs of each technique. The shorter operative time observed with the biportal endoscopic approach suggests greater procedural efficiency and convenience, which can often be associated with reduced perioperative risks, including lower rates of intraoperative bleeding, anesthesia-related complications, and reduced overall operating room utilization [49,50]. In contrast, the shorter hospital stay observed with the full-endoscopic approach may be associated with reduced tissue disruption; however, causality cannot be established from the available data [51,52,53,54]. These findings underscore the importance of tailoring surgical decisions according to the individual patient needs and clinical circumstances. Although the biportal approach may be preferred owing to its procedural speed in complex cases requiring extended operative control, the full-endoscopic approach may be better suited for patients prioritizing minimal tissue disruption and faster recovery. Further studies are needed to evaluate the long-term outcomes and patient satisfaction of each approach to refine the surgical guidelines and optimize patient care. These findings are descriptive and should be interpreted with caution, as the included studies varied in surgeon experience, case complexity, anesthesia type, and learning curve definitions. The observed differences may therefore reflect inherent confounding rather than intrinsic performance differences between the techniques.

Another notable difference lies in the choice of the anesthesia method. While the biportal endoscopic approach was performed exclusively under general anesthesia, the full-endoscopic approach could also be performed under regional anesthesia. Zhao et al. [42] performed full-endoscopic ELIF in 93 patients using local anesthesia, with epidural injections added when necessary. This may represent an important difference between the two techniques. Although no significant differences were observed in our study, it is presumed that the full-endoscopic approach is more amenable to being performed under local anesthesia [19,26,55]. Further studies are required to investigate this hypothesis and its implications.

### 4.3. How to Improve the Learning Process

**Figure 5:**
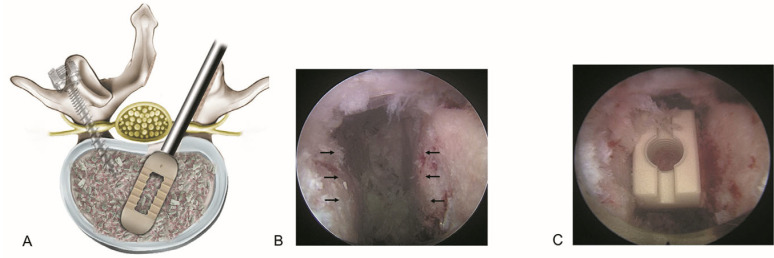
Basic concept of endoscopic lumbar interbody fusion (ELIF). (**A**). The spinal canal and intervertebral disk can be accessed via the posterolateral bypass route, minimizing irritation to the dural sac. After adequate endoscopic decompression of the spinal canal, posterolateral interbody fusion is performed in conjunction with percutaneous pedicle screw fixation. (**B**). Endoscopic view showing precise endplate preparation with selective removal of the cartilaginous endplate while preserving the bony endplate (arrows). (**C**). The final placement of the interbody fusion cage ensures optimal endplate contact to promote fusion and reduce the risk of subsidence.

Every aspiring endoscopic spine surgeon aims to learn the procedure as quickly and accurately as possible. In real-world clinical practice, general spine surgeons have few opportunities to learn endoscopy-assisted techniques during their training. ELIF represents the culmination of all cutting-edge techniques used in endoscopic spinal procedures and combines the minimally invasive advantages of endoscopic surgery with precise endplate preparation and targeted cage placement, enabling effective fusion with reduced tissue damage (Figure 5). A recent study on TLIF highlighted patient-specific and technique-dependent risks, such as cage subsidence [56]. This supports the importance of precise endplate preparation in ELIF and aligns with our interpretation that fusion-related steps may contribute to an extended learning curve.

Therefore, a systematic learning program is essential for becoming proficient in ELIF [57,58]. A structured learning program for endoscopic spine surgery may include the following three categories: (1) surgical anatomy and spatial awareness under the endoscopic view; (2) a thorough understanding of the attributes of the endoscope used; and (3) minimally invasive surgical techniques performed under endoscopy, including one-handed drilling, tissue removal using punches, and other delicate tissue manipulation.

In addition to technical considerations, consistent surgical indications are crucial for smooth learning of the endoscopic procedure. ELIF is generally recommended for typical, uncomplicated cases during the “learning” stage until the cutoff point is reached, although provisional. After achieving the “proficiency” level, the surgeon can handle more complex and severe cases. A lack of consistency in surgical indication during the “learning” period will complicate the learning curve. Variability in surgical indications during the early learning phase may substantially influence operative difficulty and performance metrics, thereby obscuring true skill progression. Early exposure to heterogeneous case profiles can prolong or distort the apparent learning curve, independent of the surgeon’s actual technical development. This factor should be considered when interpreting variability across ELIF learning-curve studies.

A typical systematic training program for endoscopic spine surgery involves several key components to ensure safe and effective acquisition of skills. These included didactic sessions to understand the anatomical landmarks and procedural steps, hands-on cadaveric workshops for practical experience, and supervised surgeries under the guidance of expert mentors [59]. Simulation-based training using virtual or augmented reality has also been proposed to enhance technical proficiency and shorten learning curves [60,61,62]. Structured feedback and regular assessments help refine surgical techniques and build confidence in endoscopic procedures. Establishing standardized training protocols across institutions can promote consistency and optimize the learning outcomes for surgeons with various levels of experience. A structured ELIF training pathway could integrate basic theoretical instruction, simulation or cadaver-based practice, and supervised early clinical cases. Incorporating these elements may help standardize skill acquisition and reduce variability in the learning curve.

However, given the small and heterogeneous evidence base, any training recommendations derived from current learning-curve data should be regarded as provisional rather than prescriptive.

### 4.4. Ongoing Learning Curve

In actual cases, three potential scenarios may follow the initial asymptote or cutoff point: (1) a plateau indicating mastery, consistent with the traditional learning curve model; (2) a rising curve reflecting the continuous acquisition of advanced skills; and (3) a declining curve associated with more challenging and complex cases. Although some studies suggest the possibility of multi-phase or ongoing learning trajectories, these patterns should be regarded as hypothesis-generating rather than definitive due to the limited evidence base [63,64]. Furthermore, some studies indicated the possibility of multiple plateaus based on different outcome measures. Based on the current literature and expert observations, the traditional notion of a single plateau may not apply, with multiple cutoff points or ongoing progression being more reflective of the learning process.

A comparison of the three groups in some studies [40,42] shows that there are multiple asymptotes, and technical skills continue to improve over time. However, identifying the actual plateau point remains a challenge.

### 4.5. Study Limitations and Future Perspectives

This study had limitations. First, the number of included studies and overall number of patients were relatively low because ELIF has not yet been widely adopted, even among endoscopic spine surgeons. With the recent surge of interest in this technique, sufficient data are expected to be accumulated in the future. In addition, institutional settings and departmental backgrounds (neurosurgery vs. orthopedics) may influence the ELIF learning curve by affecting case volume, mentorship availability, and prior exposure to MISS techniques. These contextual factors likely contribute to variability across published studies and should be considered when interpreting pooled results. Second, considerable variability in the studies may have caused bias. There were differences among the studies in the type of endoscope used (full endoscopic vs. biportal endoscopic), surgical methods (posterolateral vs. posterior), and definition of the outcome measures analyzed. However, considering that the heterogeneity among studies was low to moderate, the analysis results are relevant. Third, there were no proper explanations for the determination of the cutoff point between the early and late groups. They may have been selected randomly or based on the surgeon’s experience. This should have been determined at the asymptote of the learning curve using cumulative sum analyses. Meta-regression was considered; however, only five studies were available, making such analysis underpowered. Future meta-analyses should incorporate meta-regression as more ELIF learning curve data accumulate. Finally, the number of eligible studies was limited, and surgical techniques varied, with two using full-endoscopic and three using biportal approaches. Most studies adopted a posterolateral (transforaminal) approach, whereas only one used a posterior approach. The overall fusion rate was approximately 85%, reflecting both the technical challenges and the evolving nature of the procedure. Nevertheless, ELIF has been performed in many patients worldwide for nearly two decades since its introduction in the mid-2000s. Given its highly specific and technically demanding nature, it is timely and clinically relevant to summarize the available evidence on its learning curve. Therefore, our meta-analysis should be regarded as an early but necessary synthesis that can provide a foundation for future large-scale studies.

Previous systematic reviews on endoscopic lumbar discectomy have demonstrated that, although endoscopic technique is effective, the learning curves are steep and have not been fully standardized [47,65]. This suggests that prior experience with non-fusion endoscopic procedures may help shorten the adaptation period for ELIF. However, ELIF itself represents a technically demanding and novel application, which underscores the need for structured training programs and prospective studies to characterize its unique learning curve.

Recent advances in MISS—including robotic platforms such as the Da Vinci system—offer enhanced visualization, tremor reduction, and improved dexterity, which may help address the technical challenges inherent to ELIF. However, current applications remain early and are supported mainly by small case series, requiring further validation. These emerging tools may ultimately help shorten learning curves and improve procedural consistency [66].

## 5. Conclusions

ELIF is an emerging technique in the field of endoscopic spine surgery. Even after mastering the surgical techniques, the full-endoscopic approach generally tends to have a longer operative time but a shorter hospital stay than the biportal endoscopic approach. There appear to be no major differences in complications or fusion rates between the two methods, although definitive conclusions cannot be drawn. Observations of learning-curve progression in clinical practice should be considered exploratory rather than definitive, and structured learning programs may help support the acquisition of technical proficiency. Although this systematic review suggests that ELIF shows promising outcomes, the current evidence is still limited. Therefore, our conclusions should be regarded as preliminary, and further high-quality studies with larger cohorts are warranted to establish a more definitive learning curve and to confirm its generalizability.

## Figures and Tables

**Table 1:** Quality assessment using Newcastle–Ottawa Scale.

Study	Selection				Compatibility	Exposure			Total
	Is the Case Definition Adequate?	Representativeness of the Cases	Selection of Controls	Definition of Controls		Ascertainment of Exposure	Same Method of Ascertainment for Cases and Controls	Non-Response Rate	Quality Score (Out of 9)
Kim et al., 2020 [39]	*	*	*	*	**	*	*	-	8
Tan et al., 2022 [40]	*	*	*	*	**	*	*	-	8
Wang et al., 2022 [41]	*	*	*	*	**	*	*	-	8
Zhao et al., 2023 [42]	*	-	*	*	**	*	*	-	7
Guo et al., 2024 [43]	*	*	*	*	**	*	*	-	8

**Table 2:** Characteristics of enrolled studies.

No.	Study	Journal	Endo	Access	Anesth	Cases	Age (Years)	No. Sg
1	Kim et al., 2020 [39]	Biomed Res Int	Bi	TLIF	General	57	68.5 ± 9.4	1 OS
2	Tan et al., 2022 [40]	Front Surg	FE	PLIF	General	36	50.11 ± 9.32	2 OS
3	Wang et al., 2022 [41]	ZXFCJWKZZ	Bi	TLIF	General	55	53.19 ± 12.77	1 OS
4	Zhao et al., 2023 [42]	J Orthop Surg Res	FE	TLIF	Local	93	66.7	1 OS
5	Guo et al., 2024 [43]	J Orthop Surg Res	Bi	TLIF	General	184	65.53 ± 6.21	1 OS

Anesth = anesthesia; Bi = biportal endoscopic; Endo = endoscope; FE = full-endoscopic; OS = orthopedic surgeon; PLIF = posterior lumbar interbody fusion; TLIF = transforaminal lumbar interbody fusion; Sg = surgeon; ZXFCJWKZZ = Chinese Journal of Reparative & Reconstructive Surgery.

**Table 3:** Summary of outcome data.

No.	Study	Endoscope	Grouping	Cutoff Point	Measurements	Statistical Method
1	Kim et al., 2020 [39]	Bi	34 vs. 23	34	OT/VAS/Ambul	cum/compar
2	Tan et al., 2022 [40]	FE	12 vs. 12 vs. 12	12	OT	cum/compar
3	Wang et al., 2022 [41]	Bi	27 vs. 28	17	OT/HbL/HS	cum/compar
4	Zhao et al., 2023 [42]	FE	31 vs. 31 vs. 31	25	OT/Rad time/HS	cum/compar
5	Guo et al., 2024 [43]	Bi	29 vs. 155	29	OT/HS/Cx/Failure	com/compar

Ambul = time to ambulation; Bi = biportal endoscopic; compar = comparative analysis; cum = cumulative sum analysis; Cx = complication; Failure = surgical failure; FE = full-endoscopic; HbL = hemoglobin loss; HS = hospital stay; OT = operative time; Rad time = radiation time; VAS = visual analog pain scale.

**Table 4:** Comparison between full-endoscopic ELIF vs. biportal ELIF.

	Full-Endoscopic	*N*	Biportal	*N*	*p*-Value
Operative time (min)					
Early group	293.14 ± 85.67	43	180.98 ± 33.58	90	<0.0001
Learned group	205.12 ± 76.77	86	134.96 ± 20.95	86	<0.0001
Overall	245.23 ± 89.42	129	148.95 ± 34.46	129	<0.0001
Hospital stays (days)					
Early group	6.52 ± 1.20	43	9.36 ± 3.61	90	<0.0001
Learned group	5.32 ± 1.33	86	8.36 ± 1.86	86	<0.0001
Overall	6.05 ± 1.38	129	8.65 ± 2.55	129	<0.0001

ELIF = endoscopic lumbar interbody fusion.

## Data Availability

The data presented in this study are available upon request from the corresponding author.

## Supplementary Materials

The following supporting information can be downloaded at: https://www.mdpi.com/article/10.3390/jcm14248926/s1, Text S1: Search strategies; Table S1: PRISMA 2020 Checklist.

## Abbreviations

The following abbreviations are used in this manuscript:

CI	Confidence interval
ELIF	Endoscopic lumbar interbody fusion
MeSH	Medical Subject Headings
PLIF	Posterior lumbar interbody fusion
SMD	Standardized mean difference
TLIF	Transforaminal lumbar interbody fusion
VAS	Visual analog scale
