# Supplementary material for: The Learning Curve of Endoscopic Lumbar Interbody Fusion: A Systematic Review
Source: J Clin Med. 2025 Dec 17;14(24):8926. doi: 10.3390/jcm14248926 (PMC12733482; doi:10.3390/jcm14248926)
Supplement: Supplementary file 1 [file jcm-14-08926-s001.zip › Supplementary Materials Text S1.pdf]

Supplementary File 1. Search Strategies

Database: MEDLINE (via PubMed)
Date of search: January 27, 2025
Search strategy: ((“biportal endoscopic”[TIAB] OR “uniportal endoscopic”[TIAB] OR “full-endoscopic”[TIAB] OR “unilateral biportal endoscopic”[TIAB] OR endoscopic[TIAB]) AND (lumbar[TIAB] OR lumbosacral[TIAB] OR thoracolumbar[TIAB]) AND (“interbody fusion”[TIAB] OR Spinal Fusion[Mesh])) AND (“learning curve”[TIAB] OR “learning curves”[TIAB] OR “training curve”[TIAB] OR “training curves”[TIAB] OR "Learning Curve"[Mesh])
Results: 14

Database: Embase
Date of search: January 27, 2025
Search strategy: (‘biportal endoscopic’ OR ‘uniportal endoscopic’ OR ‘full-endoscopic’ OR ′unilateral biportal endoscopic’ OR endoscopic) AND (lumbar OR lumbosacral OR thoracolumbar) AND (‘interbody fusion’ OR ′spine fusion’/exp) AND (‘learning curve’ OR ‘learning curves‘ OR ‘training curve‘ OR ‘training curves’ OR ‘learning curve’/exp)
Results: 56

Database: Cochrane Library
Date of search: January 27, 2025
Search strategy: ((‘biportal endoscopic’ OR ‘uniportal endoscopic’ OR ‘full-endoscopic’ OR ‘unilateral biportal endoscopic’ OR endoscopic) AND (lumbar OR lumbosacral OR thoracolumbar)
AND (‘interbody fusion’ OR Spinal Fusion[Mesh])) AND (“learning curve” OR “learning curves” OR “training curve” OR “training curves” OR "Learning Curve"[Mesh])
Results: 1
